# Complementary and alternative drug therapy versus science-oriented medicine

**DOI:** 10.3205/000209

**Published:** 2015-06-23

**Authors:** Manfred Anlauf, Lutz Hein, Hans-Werner Hense, Johannes Köbberling, Rainer Lasek, Reiner Leidl, Bettina Schöne-Seifert

**Affiliations:** 1 Medical Care Centre Cuxhaven GmbH, Medical Office for Internal Medicine, Cuxhaven, Germany; 2 Albert-Ludwigs-Universität (University), Institute for Experimental and Clinical Pharmacology and Toxicology, Department 2, Freiburg i. Br., Germany; 3 University of Münster, Institute for Epidemiology and Social Medicine, Münster, Germany; 4 Wuppertal, Germany; 5 Bergisch Gladbach, Germany; 6 Ludwig-Maximilians-Universität (University), Institute for Health Economics and Health Care Management, Munich, Germany; 7 Helmholtz Research Centre, Institute for Health Economics and Health Care Management, Munich, Germany; 8 University of Münster, Institute for Ethics, History and Theory of Medicine, Münster, Germany

**Keywords:** complementary alternative therapy, CAM, science-oriented medicine, placebo research, medical ethics, homeopathy, anthroposophic medicine, phytotherapy

## Abstract

This opinion deals critically with the so-called complementary and alternative medical (CAM) therapy on the basis of current data. From the authors’ perspective, CAM prescriptions and most notably the extensive current endeavours to the “integration” of CAM into conventional patient care is problematic in several respects.

Thus, several CAM measures are used, although no specific effects of medicines can be proved in clinical studies. It is extensively explained that the methods used in this regard are those of evidence-based medicine, which is one of the indispensable pillars of science-oriented medicine. This standard of proof of efficacy is fundamentally independent of the requirement of being able to explain efficacy of a therapy in a manner compatible with the insights of the natural sciences, which is also essential for medical progress. Numerous CAM treatments can however never conceivably satisfy this requirement; rather they are justified with pre-scientific or unscientific paradigms.

The high attractiveness of CAM measures evidenced in patients and many doctors is based on a combination of positive expectations and experiences, among other things, which are at times unjustified, at times thoroughly justified, from a science-oriented view, but which are non-specific (context effects). With a view to the latter phenomenon, the authors consider the conscious use of CAM as unrevealed therapeutic placebos to be problematic. In addition, they advocate that academic medicine should again systematically endeavour to pay more attention to medical empathy and use context effects in the service of patients to the utmost.

The subsequent opinion discusses the following after an introduction to medical history: the definition of CAM; the efficacy of most common CAM procedures; CAM utilisation and costs in Germany; characteristics of science-oriented medicine; awareness of placebo research; pro and contra arguments about the use of CAM, not least of all in terms of aspects related to medical ethics.

## Zusammenfassung

Diese Stellungnahme setzt sich, auf der Grundlage aktueller Daten, kritisch mit der sogenannten komplementär-alternativen Arzneitherapie (CAM) auseinander. Aus der Perspektive der Autoren sind CAM-Verordnungen und erst recht die gegenwärtig verbreiteten Bestrebungen zur „Integration“ von CAM in die schulmedizinische Patientenversorgung in mehrfacher Hinsicht problematisch.

So werden etliche CAM-Maßnahmen eingesetzt, obwohl sich in klinischen Studien keinerlei spezifische Arzneimittelwirkungen nachweisen lassen. Die hierbei angewandten Methoden der evidenzbasierten Medizin sind, so wird ausführlich erläutert, eine der unverzichtbaren Säulen wissenschaftsorientierter Medizin. Dabei ist dieser Standard des Wirksamkeitsnachweises grundsätzlich unabhängig von der für den medizinischen Fortschritt ebenfalls essentiellen Forderung, die Wirksamkeit einer Therapie auf eine mit den Einsichten der Naturwissenschaften verträgliche Weise erklären zu können. Zahlreiche CAM-Behandlungen können allerdings auch diese Forderung absehbar niemals erfüllen, sondern werden mit vor- oder unwissenschaftlichen Paradigmata begründet.

Die nachgewiesene hohe Attraktivität von CAM-Maßnahmen bei Patienten und bei vielen Ärzten beruht u.a. auf einer Mischung aus positiven Erwartungen und Erfahrungen, die aus wissenschaftsorientierter Sicht teils unberechtigt, teils durchaus berechtigt, aber unspezifisch (Kontexteffekte) sind. Mit Blick auf das letztgenannte Phänomen halten die Autoren den bewussten Einsatz von CAM als verdeckte therapeutische Placebos für problematisch. Zudem plädieren sie dafür, dass die Schulmedizin sich wieder systematisch um mehr Zuwendung, ärztliche Empathie und den maximalen Einsatz patientendienlicher Kontexteffekte bemühen müsse.

Die nachfolgende Stellungnahme diskutiert nach einer medizinhistorischen Einleitung: die Definition von CAM; die Wirksamkeiten häufigster CAM-Verfahren; die CAM-Inanspruchnahme und -Kosten in Deutschland; die Charakteristika wissenschaftsorientierter Medizin; die Erkenntnisse der Placebo-Forschung; die Pro- und Kontraargumente zur Anwendung von CAM, nicht zuletzt unter medizinethischen Aspekten.

## Introduction

The Drug Commission of the German Medical Association published an opinion for evaluating methods of medical therapy “outside scientific medicine” in 1998 [1]. Since then, this topic, which affects central issues as to the appropriate standards and criteria of pharmacotherapy has become even more important. This is shown by the ongoing and controversial discussions in public and in different committees and professional organisations. Therefore, a work group of the Drug Commission has decided for an updated opinion. 

Medical treatment has been committed to the well-being of patients ever since the start of medical science. The underlying notions of ailments and their treatment options have undergone numerous changes in the course of time. Although the significance of experimentation and reliance on mathematics for science was already known in the 13^th^ century (Roger Bacon) and ‘new thinking’ that was committed to empirical verifiability of therapy successes, and scientific research was also advocated in medicine in the 16^th^ century with the Enlightenment (René Descartes and Francis Bacon), antique and mediaeval notions of ailment and cure with Galenic humoralism, mythical-spiritual and symbolic meanings were still relevant for medical therapies until the 19^th^ century. In retrospect, treatment successes of doctors of those times are predominantly regarded as contextual (placebo) effects. Except for a few individual cases, like the comparative use of different foods for scurvy in a controlled trial by James Lind (1747), questions were not asked for testing the efficacy of medicinal products in those days [2]. It was only in the 19^th^ century that the required conditions for systematic evaluation of the efficacy of therapeutic interventions were achieved by a modern understanding of ailments and their classifications and the inclusion of statistical methods in evaluating treatment results. Another century however went by until the methods of what we today call evidence-based medicine (EbM) had been established to estimate the benefit for patients, largely independent of the kind of therapeutic intervention. Although there are undeniable successes of modern scientific medicine today and assessments of therapeutic interventions have become the standard for the approval and use of medicinal products, many patients and doctors or other therapeutists are advocates for so-called complementary and alternative medicine procedures, whose use can mean adherence to or a ‘recidivism’ into pre-science from the view of science [3]. 

Therefore, complementary and alternative medicine procedures as well as contextual (placebo) effects in the pharmacotherapeutic practice are subsequently critically discussed based on current studies and data.

## Definitions

### Complementary and alternative medicine procedures

The term “Complementary and Alternative Medicine” has been accepted internationally and is also used here with its abbreviation “CAM” [4]. [The German legislator uses the term of “special therapy approaches” for the medicinal groups primarily discussed below.] 

According to an expert estimate [5], currently about 400 CAM procedures are practiced, that vary from anthroposophy and aromatherapy via Bach flower therapy, homeopathy, naturopathy, phytotherapy all the way to Tai Chi and Yoga. An indisputably positively formulated definition of CAM appears impossible. None of the positive criteria for characterisation strived for in [6], [7], [8] (see also Table 1 (Tab. 1)), apply to all complementary medicine procedures uniformly, since their methodical approaches partially overlap or contradict each other.

Depending on the perspective, the procedures concerned are not always given the same collective headings (see Table 2 (Tab. 2)). Some of them primarily stress the nonconformist origin or methodology; others the comparably limited side effects (“softness”), even others place the “holistic” approach of CAM measures in the foreground. Finally, other terms underline the distance to science-oriented medicine and try to devalue the latter with the term “academic medicine” (“Schulmedizin”).

**Figure 1:**
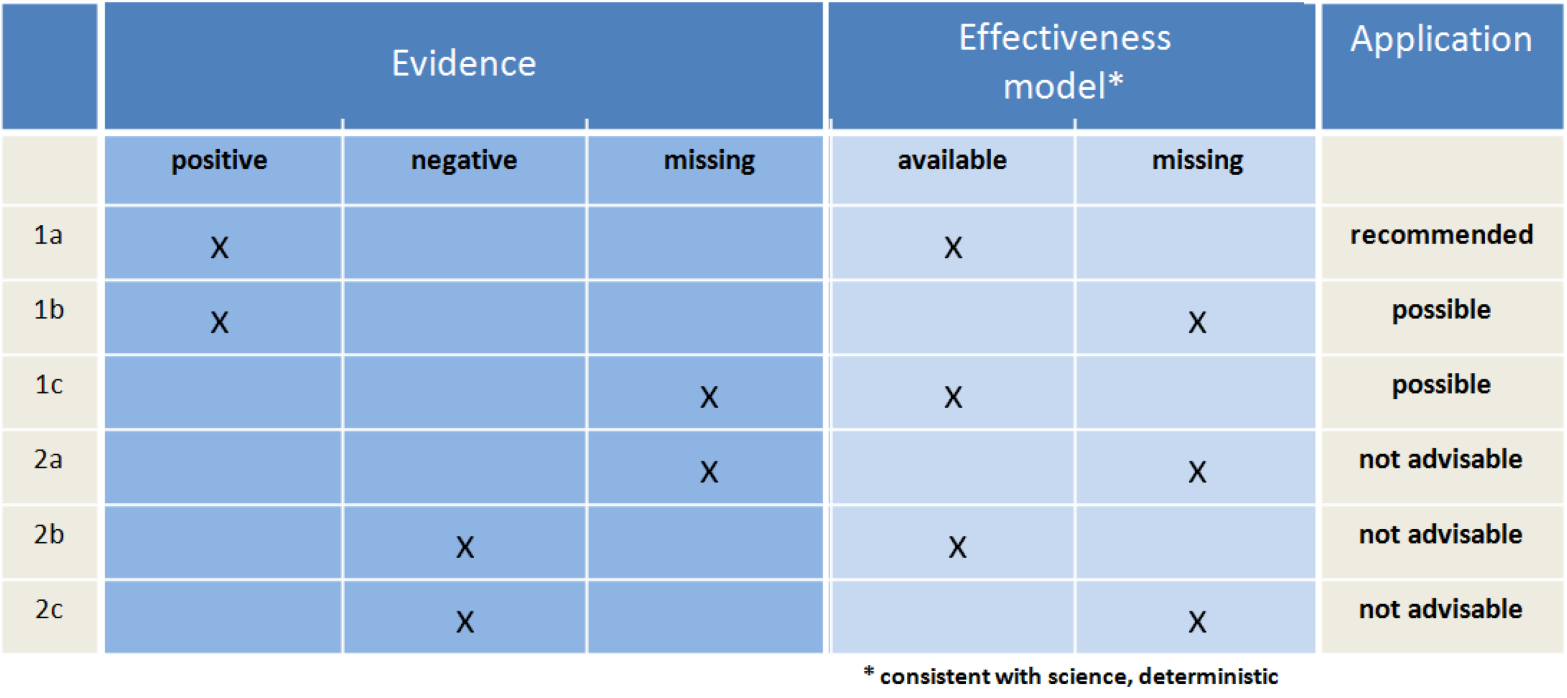
Recommended use and justification of therapies in medicine [19]

CAM procedures can primarily be defined via their distance to science-oriented medicine. This is also intended by the majority of their exponents. Several CAM advocates in fact appear not to be interested in scientific explanation and testing with a view to the mentioned procedures, and are not ready to question their own therapeutic or diagnostic strategies if they do not prove to be successful (see Figure 1 (Fig. 1), therapy group 2c). Exponents of some procedures also principally abstain from testing their therapy forms; others dispute the applicability of statistical procedures with reference to the required individualisation of their therapeutic strategies (see Figure 1 (Fig. 1), therapy group 2a). On the other hand, the need for research is also increasingly being called for even from the CAM-side and reference is being made to already available test results from clinical studies, systematic reviews and meta-analyses [9], [10], [11], [12]. However, even if these studies and systematic reviews do not certify any relevant therapeutic effect to the medicinal CAM procedures, often no conclusions are drawn about their use. The attempt to achieve acceptance through scientific seriousness is a clear loser to lobbying which takes place at the national and international level [8], [9], [10], [13].

In summary, thus, the CAM definition, be it from the internal or the external perspective, is dominated by two important aspects. One stresses the soft or “nonconformist” origin or methodology, the other the “anti-scientific” basis of the procedures accompanying this camp. In this background, the suitable choice of terms already poses a significant problem [14]. Understanding CAM as that domain of treatment which is outside science-oriented medicine according to the self-concept of its exponents and opponents is offered as a pragmatic solution. This definition represents the predominant parlance today and is also used by us subsequently. Thus CAM remains an indistinct (fuzzy) heuristic term. It is basically not to be precluded that measures attributed today to CAM may tomorrow belong to science-oriented medicine after corresponding proof of efficacy. Such a switch from individual CAM procedures to science-oriented medicine would not however prove the therapeutic value of other CAM procedures, leave alone all CAM procedures.

### Academic medicine as science-oriented medicine

The convictions that the onset and treatment of ailments are principally explicable causally as part of a scientific view of the world, and that treatment successes must be able to be generalised and measured, belong to the basic self-concept of contemporary conventional medicine. This requirement for coherence of explanation and methodological rationality however in no way reduce medicine *itself* to a science. Rather, it is primarily involved in the interests of patients according to extensive opinion as a “practical science” [15], [16]. It uses scientific methods and insights, which are not limited to the sciences, but also include psychology, social sciences or anthropology for promoting itself. In addition, it requires value orientations, serves cultural expectations and follows social understanding, which may themselves not be scientifically justified. Since treatment with and in medicine ever so often needs to be done *without* having satisfactorily (validated or even scientifically explained) ready therapies, etc., it would be exaggerated to speak of existing academic medicine as “scientific medicine”. The latter is in fact an ideal, to which academic medicine is oriented. This should therefore more correctly be designated as “science-oriented medicine” and thus should not refer to each and every one of its exponents and certainly not to each of its practices, but to the undertaking with its basic orientation. 

Even when there are ongoing controversies among scientific theoreticians about what precisely is to be understood as science, there are consensual minimum standards. This scientifically minimum standard for medicine, as practical science, exists in four basic assumptions which should be specified in greater detail below. 

**(I):** The first feature of scientific medicine consists in the requirement of being able to prove empirically that medical interventions (here medicinal products) provide clinical benefit for patients. More precisely, this means proving treatment successes in both patient-relevant target dimensions of the life extension or improvement of the health-related quality of life (due to positive influence of the condition and complications) as well as the absence of intolerable undesired effects. In individual cases, such dramatically high and usually life-saving effects may occur with new treatments, that their validation does not require any further controlled test conditions and it would not be ethical to represent them (by way of example: insulin for diabetic coma, vitamin B12 for perniciosa, streptomycin for tuberculous meningitis, penicillin G for streptococcus infections) [17]. However, proof of benefit is normally obtained by gathering and evaluating aggregated patient data in clinical studies using suitable biometric methods. Thus, chances of benefit and risks of harm are identified, strictly speaking. How a therapy works is not supposed to and cannot primarily be answered by such stochastic experiments (e.g., randomised controlled trials), rather only the fact that it does work [18]. These experiments with random allocation of treatment options contribute to resolving the question of whether the change in the course of an illness occurred only in temporal correlation with a treatment (post hoc) or is also causally determined by it (propter hoc).

As controversial as individual questions may be when choosing evidence specifications within science-oriented medicine, this fact does not in the slightest affect their basic requirement for critical evaluation of medicinal measures according to the best possible standard. In addition, it is required to draw consequences from such outcomes for therapeutic recommendations. This in particular means excluding therapeutic procedures if they prove to be ineffective or too harmful.

**(II):** Systematic endeavour towards rational explanations for the development, prevention and treatment of ailments is the second essential feature of science-oriented medicine. According to its self-concept, understanding medically relevant cause-effect relations using theory formation and systematic experimental backup of hypotheses is neither just a wish nor one of the several possible ways to effective medicine. This endeavour is much rather supposed to act in favour of a step-wise improvement of medical practice and is a part of an educated world view that considers medicinal phenomena and not the least therapeutic successes to be understandable in principle. In addition to its main requirement for empirical validation of medicinal interventions, scientific medicine rejects therapy forms which contradict the basic knowledge of sciences, as is the case with homeopathy. 

The basic credo expressed in (I) and (II) applies regardless of the fact that even today there continue to be many ailments which cannot be explained at all or can be explained only unsatisfactorily, and that there are numerous controversial views within science-oriented medicine. After all, a large part of treatment rules of scientific medicine are characterised by the fact that they can rely on plausible, although falsifiable deterministic models and stochastic results. It should be stressed once more that the deterministic and stochastic paths to knowledge are principally independent of each other, although there is a strong correlation between the two of them. Model assumptions do not commonly prove themselves in stochastic therapeutic attempts. On the other hand, stochastic experiments may be successful before an adequate model assumption exists for the efficacy mechanism of a therapy.

With a view to CAM and placebo treatments (see below), it should be stressed that the stochastic path to knowledge gives even such medicinal products an option to prove their efficacy for which there are no efficacy models with fundamental scientific hypotheses. Efficacy tests that rest upon evidence-based medicine do not basically require any explanation models for the effects to be tested.

**(III):** There can be no adequate alternatives to scientific medicine. Scientific pharmacotherapy does not need any addition or complement. Scientific medicine is developed by elimination of therapy forms which lack efficacy or have unacceptable risk/benefit ratios and while integrating new methods of different origins. 

In summary, based on (I)–(III), there are recommended uses for therapeutic procedures (Figure 1 (Fig. 1), [19]). Firstly, procedures with a plausible efficacy model and positive evidence (1a of the Figure) are recommended. A possible application may arise if the evidence is positive even if a plausible efficacy model is (still) missing (1b), just as in case of a plausible efficacy model but (as yet) missing evidence (1c), a not uncommon status in science-oriented medicine. Decision criteria are the lack of alternatives as per 1a and therapeutic exigency. On the contrary, therapy forms with negative evidence regardless of the plausibility of the efficacy model (2a, 2b) are advised against, as are those with a missing model and missing evidence (2c). [The confusion due to the linguistic closeness of negative evidence (= there are valid studies with negative outcomes) and lack of evidence (= there are no valid studies) needs to be noted.] 

**(IV):** In addition to its self-commitment to a scientific basis in the choice of its methods, medicine is a patient-related, practical discipline [20], [21]. This requires consideration of psychological, sociological and economic insights. In particular, one wonders to what extent the development of modern medicine considers the postulated unity of body and mind and is in line with its patient orientation. Medical experience is not only in agreement with the fact that bodily ailments have an entire spectrum of accompanying spiritual symptoms and consequences, but also with the fact that this spectrum may vary considerably with the same bodily ailment. Accompanying spiritual symptoms and consequences of bodily ailments thus also require “understanding insight” just like bodily dysfunctions [22].

Modern science-oriented medicine commonly does not comply with this dimension despite this undisputed medical insight. The currently applicable rules of economisation contribute significantly to this. The time pressure under which many doctors need to work, the relative disregard for “talking medicine” in the education and reimbursement system and the dominance of technical methods are viewed negatively by many patients. Disregard for personal attention, individualised view, non-technical perspectives of individual patients and their very own illness experiences may lead to deficits and make science-oriented medicine unattractive in the view of patients.

## Complementary and alternative medicine procedures of drug therapy – a closer look

### Therapy forms and approaches with special legal status

In Germany, phytotherapeutic, homeopathic and anthroposophic procedures are more commonly used than in other countries. This is explained, among other things, by the exceptional legal position for so-called “Special Therapy Approaches”, which were introduced in 1974 in the German Medicines Act (AMG) and in social legislation (SGB V). In addition, health insurers have been legally allowed to offer non-prescription pharmaceuticals, but only those available at the pharmacy, remedies and aids and services of unauthorised service providers such as “optional benefits” since 2012, as long as they were not excluded by the Joint Federal Committee (Gemeinsamer Bundesausschuss). Many insurers are using this option due to competitive reasons, among others. Compared with pharmaceuticals of science-oriented medicine, for which valid results of controlled studies have to be submitted, the aforementioned CAM procedures are thus allowed simplified approval provided they are endorsed in the internal consensus of committees of their own therapy approach (“Binnenanerkennung”). The “explicit consideration” of these therapy approaches is justified by the German Federal Institute for Drugs and Medical Devices (Bundesinstitut für Arzneimittel und Medizinprodukte) with “scientific pluralism in the area of drug therapy” [23], as if it involved selecting the offer best suited to the context from among theoretically equivalent offers. Scientific and non-scientific can however not be combined as elements of a “scientific pluralism” due to logical semantics.

### Phytotherapy

Herbal medicinal products historically belong to the longest used medicinal products and are a part of many traditional medicinal approaches in folk medicine not only in Europe, such as Traditional Chinese Medicine (TCM) or traditional Indian medicine (Ayurveda). Originally, only unmodified plants or plant parts were used for treatment, which may have partly been explained practically, and partly corresponded to antique and mediaeval notions, according to which plants as a whole had qualities which promote healing. The hope already articulated in the 14^th^ century by Paracelsus of isolating the “Quinta essentia” which promote healing from the entire plant was only implemented in the 19^th^ century with the pure presentation of plant contents which are even today used as medicinal products.

With this, it was possible to employ a chemically identified substance in defined and reproducible dosage without simultaneous administration of accompanying substances which were unnecessary or possibly caused side effects. Examples for chemically defined substances of established pharmacotherapy, developed from historical phytotherapy, are for example isolated morphine in 1805 and artemisinine, a more recent anti-malaria drug [24], [25]. They are however no longer designated as phytotherapeutics in the strict sense of the word due to their scientific validation [26].

Herbal medicinal products, on the contrary, consist, in the actual sense of the word, of preparations of entire plants or herbal parts and are thus always mixtures of many substances. With the intention of organising phytotherapy more rationally, industrial plant extracts standardised on the basis of active or main substances are predominantly used today (so-called “Rational Phytotherapy”). Evidence often stated for confirmation of the efficacy of phytotherapy as therapy approach generally relate to such preparations. Thoroughly positive efficacy evidence exists for some of them, e.g., Colchicum (late bloomer) extract for acute treatment of gout [27], [28], [29]. For others, like mistletoe preparations, no conclusively recognisable proof for the benefit in cancer treatment could be ascertained until now (see below). Overall, however, the database is very limited even for monoextract preparations, be it due to the bad quality of studies or the fact that the companies manufacturing preparations, which are not subject to patent protection, have only limited interest in extensive tests for relevant clinical endpoints [30], [31].

Besides ascertained proof of benefit, data about the safety or harmlessness is additionally often lacking for herbal medicinal products. Because, contrary to the widespread notion that the natural origin of herbal preparations guarantees their “soft” effect, herbal medicinal products can be accompanied by considerable side effects (e.g.: market withdrawal of Kava Kava due to hepatotoxicity, nephrotoxicity of aristolochic acid) or interactions (e.g., hypericum preparations) and in addition may hide significant risks due to variations in authenticity, purity and active ingredient content [32], [33], [34], [35], [36], [37].

The database for probabilities of benefit and risks of harm of so-called traditional herbal medicinal products include those of Far East origin which are partly propagated as a recourse to antique and mediaeval spiritual or popular medicinal notions (e.g., “Hildegard medicine”, Ayurveda). In this regard, a treatment with mixtures of many substances from which only one or some are responsible for the effect, while the others are dispensable or entail potential risks is considered to be a historically established therapy form which nonetheless requires substantiation [16]. Exponents of these procedures however propagate that a curative plant could be more than the sum of its contents (synergy of effects, neutralisation of side effects), which would however need to be concretely proved from the perspective of science-oriented medicine. 

### Homeopathy

Homeopathy was developed by Samuel Hahnemann at the end of the 18^th^ century, thus at a time of progressive medical-scientific theory formation, but with as yet predominantly pre-scientific practices in actual patient treatment. Thus, the frequently practiced blood-letting or the administration of herbal or mineral remedies from the still predominantly mediaeval apothecary was usually ineffective and not rarely fraught with drastic side effects. In this background, the preference of many patients at that time for the new “soft” treatments, which precluded such side effects, was thoroughly understandable. However, the basic principles of homeopathy could not be explained plausibly even according to the scientific standards existing at that time. 

These requirements for explanation were the cause for criticism then and in subsequent decades not only by science-oriented doctors, but also by “unorthodox” supporters of homeopathy. Still, homeopathy won sympathy among large parts of the population, especially in influential circles of bourgeoisie and noblemen, which certainly contributed to its societal legitimacy and also to the later national promotional measures [“New German Medical science” (“Neue Deutsche Heilkunde”), “Internal Recognition” in the 2^nd^ SHI Reorganisation Act (GKV-Neuordnungsgesetz) of 1997].

Currently, the undisputed popularity of homeopathy and other CAM procedures, as it can be determined in surveys (e.g. [38]), has been elevated to the level of surrogate parameter for its efficacy. But the actual core question of whether the positive effect of these therapy forms are sufficiently proven mostly remains unanswered in public presentations. Yet, it is precisely due to the fact that the basic principles of homeopathy, i.e., the “simile principle” (like heals like) or the “potency/dynamisation” procedure, cannot be explained scientifically that a stringent proof of efficacy is especially required.

Hahnemann’s drug tests were not efficacy tests, but determinations of drug symptoms (“drug image”) on healthy people, which were often conducted as self-experiments [2]. Neither did subsequent drug examinations on the order of the national socialist Reich’s Ministry of Health nor did corresponding investigations by Paul Martini, who was sceptical about it, provide relevant differences to placebo administration [39], [40], [41], [42], [43], [44]. Whereas only few and sporadic observations are available on the therapeutic efficacy of homeopathic products from the time before the 2^nd^ World War, numerous clinical studies have appeared since, which are however often unsatisfactory methodically. Evaluating these studies with methods of evidence-based medicine, thus by meta-analyses and systematic reviews did not result in any superiority of homeopathic products over placebo administration after a high degree of international consensus [45], [46], [47], [48], [49], [50], [51], [52], [53], [54], [55], [56], [57], [58], [59], [60], [61], [62], [63], [64], [65], [66], [67].

Since this alternative medicine therapy approach plays a significant role in practice and in public observation, a few summaries will be cited. Thus

- the Drug Commission of the German Medical Association wrote in its opinion of 1998: “Since over 140 years of existence of and experiences with homeopathy including the evaluation of its results with modern meta-analyses were not capable of making its efficacy probable … it raises concern, if further costly studies are still required, instead of drawing consequences from present knowledge” [1], similar to [68].
- the House of Commons of the British Parliament in the year 2005: “… there is no credible evidence of efficacy for homeopathy, which is an evidence based view. … To maintain patient trust, choice and safety, the Government should not endorse the use of placebo treatments, including homeopathy. Homeopathy should not be funded on the NHS and the MHRA should stop licensing homeopathic products” [69].
- the National Center for Complementary and Alternative Medicine in the year 2013: “Most rigorous clinical trials and systematic analyses of the research on homeopathy have concluded that there is little evidence to support homeopathy as an effective treatment for any specific condition” [70].
- and most recently the Australian Regulatory Institution National Health and Medical Research Council (NHMRC): “Conclusions: Based on the assessment of the evidence of efficacy of homeopathy, NHMRC concludes that there are no health conditions for which there is reliable evidence that homeopathy is effective” [71].

### Anthroposophic medicine

Anthroposophic medicine is based on the spiritual philosophy of life of its developer Rudolf Steiner (1861–1925). It looks upon itself as scientific extension of medicine to dimensions of the spiritual world, whose discovery goes beyond the one-sidedness of just the knowledge of nature. Only the physical body is accessible to scientific knowledge from amongst the postulated levels (“elements of being”) of physical body, etheric body, astral body and ego-organisation. The other levels, on the contrary, required the development of especially capable people through imagination, inspiration and intuition. Illness and its cure are understood as a disturbed and respectively restored relation of the above-mentioned elements of being.

The use of anthroposophic remedies, which can be of vegetal, mineral or animal origin, is defined by the affinity between human beings and nature and is supposed to exert an influence on the mentioned elements of being. The application of mistletoe for tumour therapy is founded on phenomenal analogy (parasitic autonomous growth in mistletoe like tumour) and is supposed to lead to the transmission of etheric forces from the tree and mistletoe onto the human being. Since Steiner explicitly rejects the experiment as basis of knowledge for drug therapy, it is not astonishing that the measurability of anthroposophic therapies with the instruments of evidence-based medicine is often disputed by its exponents and that there are only insufficient efficacy studies for anthroposophic remedies [72], [73], [74], [75], [76]. This also applies for the treatment of tumour patients with mistletoe preparations, for which there do exist controlled trials, but neither an effect on the tumour progression nor on the survival time is ensured, while references to a possible improvement of the life quality in breast cancer patients require testing [77], [78], [79], [80]. 

Seen scientifically, there is no reason to deviate from the evaluation of the German Medical Association (Bundesärztekammer) in summaries, which writes in its memorandum of 1993 on medical treatment as part of “special therapy approaches”:

“It is characteristic for objectively effective treatment procedures that they are compatible with the generally recognised notions of aetiology and pathogenesis of ailments and are based on a concept that is supported either experimentally or by the independent, reproducible experience of the respective therapeutists. This does not correspond to the procedures of special therapy approaches of homeopathy and anthroposophy.” [39]

### Use

Systematic investigations of these questions were first published in 2002 in the report “Utilisation of alternative methods” in medicine [81] which appeared as part of the health monitoring of the federation and subsequently in model projects of some state health insurers [82]. The projects and surveys conducted since the mid of the 1990s resulted in the fact that just about three-quarters of adult Germans had had experiences with nature cure remedies, and that this percentage was clearly higher than at the time of comparison in 1970 [81]. The unanimous conclusion was that the use of CAM procedures was much more common in women than in men and moreover that it was positively correlated with the increase in the level of education. The 2002 Health Monitor confirmed the increasing popularity of alternative medicine and identified similar figures [83]. A representative study showed that about 70 percent of women and 54 percent of men in the age group of people aged between 18 to 69 years had used at least one CAM procedure in the twelve months before the survey. The most common related to phytotherapy, homeopathy and acupuncture and higher education was correlated to higher degree of use [84]. Similar results were provided by comparable questionnaires for the city of Lübeck [85] and for the members of a private health insurance [86], [87]. CAM procedures like homeopathy and phytotherapy play a role even in children, as shown by a study on two German cohorts published in 2012. Thus, 24% of the examined children had taken homeopathic medicinal products and 11.5% had taken phytotherapeutic medicinal products in the four week period prior to the survey, where the latter utilisation was positively associated with a higher level of education of the mothers [88]. 

The 2002 Health Monitor already addressed the patient perspective on CAM [83]. It was shown that women were often more sympathetic to complementary medicine than men; similar findings were observed for human beings with a higher level of education, while age differences hardly had any influence. Self-classification of one’s own health condition hardly appeared to have any significance. Whosoever indicated that they were very strongly concerned with their health on a day to day basis were clearly also supporters of CAM methods more often. Personal experiences with these procedures exerted a positively reinforcing effect more or less independent of whether the hoped medicinal success took place or not. 

This latter finding is ascribed to the fact that CAM procedures and CAM therapeutists create effects which cannot be ascribed to clinical benefits in the strict sense of the word, but rather to meeting the needs for communication, to social and emotional support and even to give sickness a meaning. Thus, positive features of CAM measures were stressed to be the fact that causes of spiritual illness were included and that therapeutists took more time for the patients. However, many people surveyed simultaneously also pointed out to the risks and limited indications of CAM procedures which were to be used especially in unclear or long drawn-out conditions. The fact that CAM could compete or even replace science-oriented medicine did not appear meaningful to most of them [83]. Other representative surveys displayed similar results [84], [85], [86], [87]. 

Comparable data are reported by newer doctor surveys. Thus, about 60 percent of doctors surveyed in a representative general practitioner survey conducted in 2009 reported that they use CAM procedures regularly in their practice [89]. Similar results that 51 percent use CAM methods [90] were provided by a telephone survey of 516 German general practitioners published in 2008. 

### Costs

**Figure 2:**
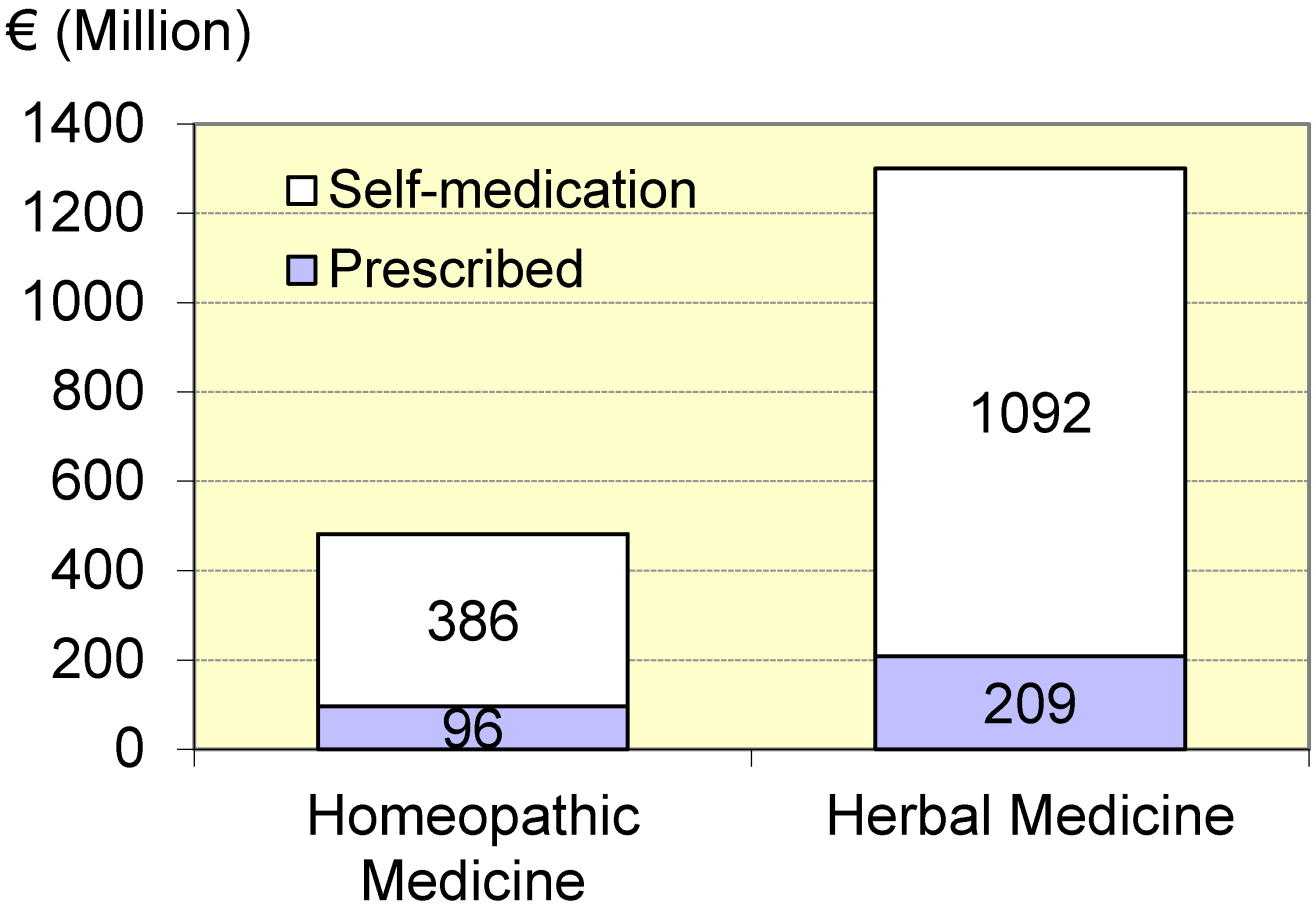
Pharmacy revenues for homeopathic and herbal medicinal products in Germany in 2013, information of the German Medicines Manufacturers’ Association (2014)

**Figure 3:**
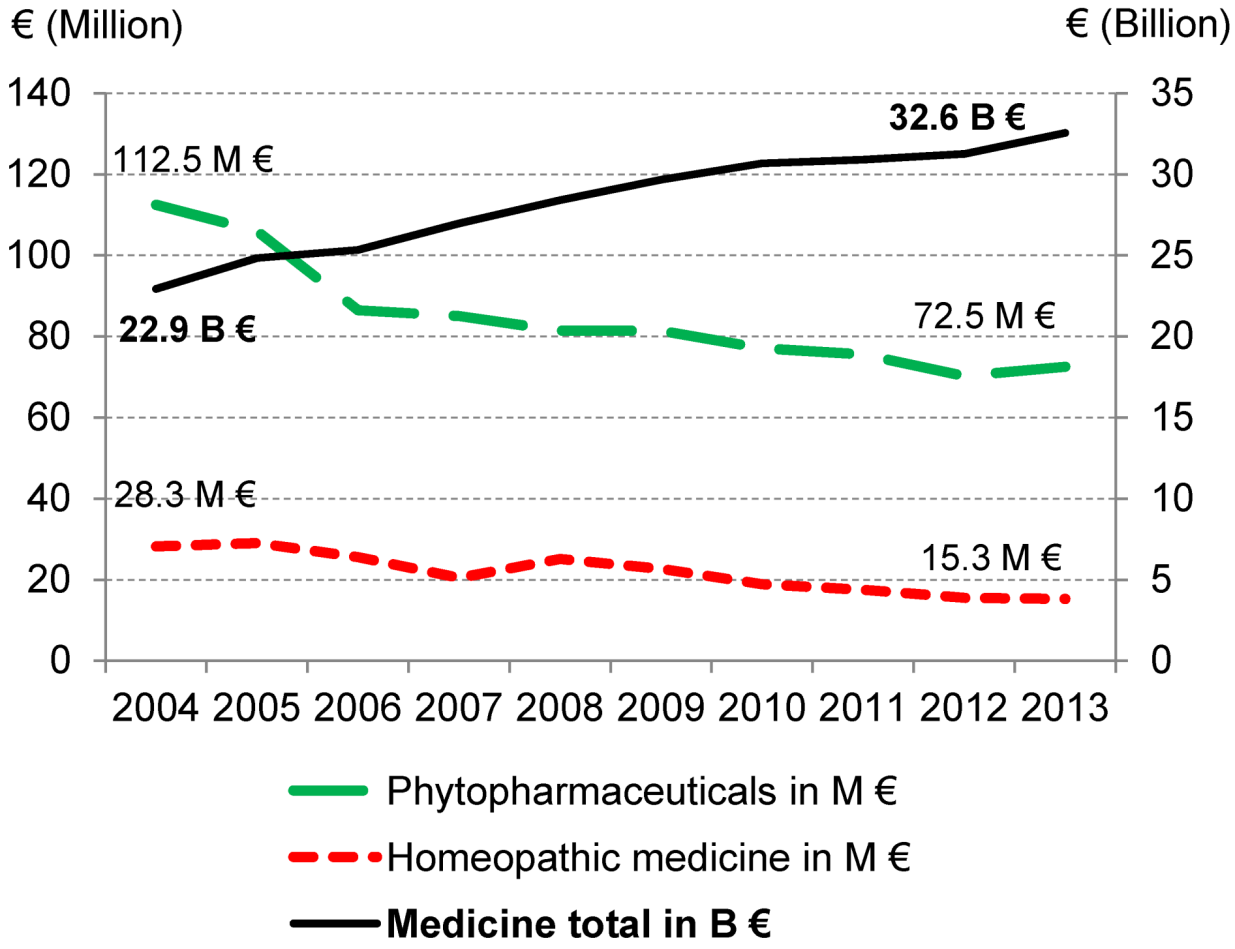
Benefit payments of statutory health insurance (SHI) for homeopathic products and herbal medicinal products as well as pharmaceuticals overall during 2004–2013, information of the German Pharmaceutical Industry Association, different years

The German Medicines Manufacturers’ Association (Bundesverband der Arzneimittelhersteller) regularly reports about apothecary sales of homeopathic and herbal medicinal products. Accordingly, in the year 2013, 1.78 billion Euros were spent for both categories together, where just about three quarters of the payments were allocated to herbal medicinal products (Figure 2 (Fig. 2)). A doctor’s prescription was submitted in a fifth of the earnings with homeopathic medicinal products, in about a sixth with herbal medicinal products. However, a doctor’s prescription does not automatically lead to an assumption of payments by the statutory health insurance (SHI). According to data of the German Pharmaceutical Industry Association (Bundesverband der Pharmazeutischen Industrie), the SHI assumption of payments has been decreasing for both drug categories since 2004 (Figure 3 (Fig. 3)); the percentage of both of them in the drug payments of SHI overall, which rose considerably, thus has fallen to less than half since 2004 and was 0.27% in 2013 [91].

However, in this statistic, the expenses for homeopathic and herbal medicinal products are not included as optional benefits (since 2012) and administrative modifications cannot be excluded. Overall, the expense of SHI due to both drug categories constitutes a very small percentage. Moreover, private health insurers paid just about 40 million Euros in the year 2011 overall for medicinal products of the “special therapy approaches”. From the total expenses for medicinal products of private health insurance (PHI), around 1.1% are allocated to homeopathy, 0.5% to anthroposophy and 2.7% to phytotherapy [Scientific Institute of PHI (Wissenschaftliches Institut der PKV); personal communication]. The predominant share of CAM costs is borne privately. 

## Placebo treatment: observed in greater detail

### General information

Psychosocial and contextual factors can play a big role in the therapy success of a medication. Medical history and the daily experience of doctors and evidence from scientific investigations show this. Expectations of patients, doctor’s empathy and attention as well as the therapeutic ritual of medical prescription and application belong to the important factors in this regard. The therapeutic main role is in fact attributed to them in case of placebos without active drugs. This can also be assumed for the predominant part of CAM therapies.

There is a broad consensus within the medical scientific community that placebos are allowed to be used under specific conditions in biomedicinal research, more precisely in the control groups of clinical studies, even if some details are disputed [92]. On the contrary, there are principally very controversial opinions about the justifiability of placebos in therapeutic practice. Pursuing with this subject thus appears to be important where not only “pure” placebos (without any active substance), but even so-called “impure” or “pseudo placebos”, need to be involved, i.e., actual drugs which are however not specifically effective for the respective illness condition when considering existing and justified practices. 

Since the highly acclaimed work “The Powerful Placebo” by Henry Beecher in 1955, the assumption that in many conditions about 30 % of the patients are responsive to a placebo administration or that it is responsible for more than 30 % of the observed effects is widespread [93], [94], [95], [96].

In this regard, several other questions which are dealt with by pharmacology in researching, developing and clinically using drugs arise: Which diseases do placebos work for? How do placebos work? Is there a uniform work mechanism or are there different ones? Who responds to placebos? Can their effect be predicted and reproduced? How long do placebos work? Does the placebo effect depend on dosage? Do placebos have undesired side effects? How often are placebos used in daily practice?

### Efficacy contexts

Placebo effects were described in treatment attempts of very different diseases which accompany psychological strain, especially aches, but also anxiety, depression, Parkinson’s disease, gastro-intestinal discomfort or angina pectoris [97], [98], [99]. In this regard, clinical end-points to be collected subjectively, like pain or sensitivity, appear to be particularly “placebo sensitive” [96], [98]. This does not preclude making physiological correlations objective, such as EEG changes, e.g., in case of improvement in insomnia. On the contrary, placebos do not demonstrate any or only comparatively low efficacy to objective illness-specific (“hard”) end-points like tumour growth or survival time [100]. Patients under anaesthesia or Alzheimer patients with difficult cognitive deficits also demonstrate no to clearly weakened placebo responses [101], [102]. These findings suggest that placebo effects operate via consciousness and influence the feeling of illness more than the illness itself [96], [98], [103], [104], [105]. 

The specific drug effect, but not the placebo effect itself, can be determined in normal two-armed clinical comparative studies, with a verum and a placebo group, since it cannot be separated from other context effects and distortions (see below). Quantifications of the placebo effects on the basis for comparison of verum effects thus lead to error and are at best of limited practical value. In order to identify the actual placebo effect, the two-armed study may be inserted into a third arm, in which the patients obtain no treatment. The placebo effect then possibly consists of improving the group treated with placebo in contrast to untreated patients [106]. Low to moderate effects of placebo administration were found in an extensive Cochrane review containing 200 such studies on different indications, especially in studies with continuous and patient-reported end-points, such as in case of pain, for example [94], [107], [108], [109]. However, the administration of placebo is, otherwise than often assumed, not associated overall with a general and clinically significant advantage, according to the summary. Patient-reported effects, for instance about influencing aches are prone to error, very variable and dependent on context. Another meta-analysis of the same studies by another author group determined that verum therapy was only considered superior in the 37 studies with binary end-points of the placebo treatment, which did not apply to the 111 studies with continuous end-points [110]. 

A procedure for calculating the placebo share of a treatment effect is the application of drugs (or even a placebo) in open versus hidden form. E.g., analgesics can be administered by an automatic infusion pump for this purpose, at times with and at times without the current knowledge of the (informed consent giving) patient. Analgesics work in both forms, with open and hidden application, but the dosage required for hidden administration for equivalent pain inhibition is significantly higher [111].

Such observations make it clear that alleged “placebo effects” can be explained less by individual factors such as the administration of a placebo medicine and much rather by aspects of the overall treatment context. The expectations of the patient, doctor’s attention and empathy, type and scope of the education about the measure or kind and form of applying the placebo are included herein (see also Table 3 (Tab. 3)) [98], [112]. Several other phenomena may just simulate a placebo effect, e.g., spontaneous improvement, regression to the mean and personal features or behavioural changes while participating in a study [95], [98]. Thus, the supposedly observed placebo effect may be composed of a true placebo effect, other non-specific effects and statistical distortions in different forms [106].

### Effect mechanisms

Since placebo effects are missing in unconscious patients and diminish in case of hidden application (see above and [101], [102] they can be understood as psychophysiological reactions to factors of the treatment context. Two psychological mechanisms are essentially postulated as the basis for such placebo effects: expectation and conditioning [113], [114]. According to the expectation theory, the patient is prepared for a positive effect of the placebo, since this was explicitly projected to them (“I am now giving you a strong remedy against your pains”) or is to be expected in the treatment context. Moreover, phenomena of the traditional conditioning according to Pawlow can prepare a placebo response: If the patient has had positive experiences during previous treatments with an analgesic, thus a pain reliever, this can trigger a conditioning. If the effective analgesic tablets had a certain colour or taste, these characteristics can act as a conditioned stimulus, and result in pain reduction as a placebo even without an active agent [113], [114], [115]. The more often both the attractions, the positive effect of a verum treatment and the conditioning attraction of the method of application (tablet properties, injections, etc.), are combined, the higher the probability that the placebo will later have the same effect as the verum. These principles may apparently also apply to immunological drug reactions if only the taste of a verum is imitated by a placebo, for example [116], [117]. The fact that placebo effects also occur in invasive therapy procedures, e.g., acupuncture or arthroscopic interventions, is only mentioned in passing as part of these medicinal therapeutic considerations [118], [119].

### Mechanisms of the placebo effect with aches

Levine was the first to show in 1978 that endogenous opioids can play a role in the analgesic effect of the placebo [120]. Thus, the analgesic placebo effect was nullified by the administration of the opioid receptor antagonist naloxone. These results were later confirmed and broadened [120], [121]. Recently, Benedetti has additionally proved that the analgesic effect of placebo is dependent on the activation of the endogenous cannabinoid system [122]. The conditioning of the subjects allowed continuing a previous positive experience of taking a pain reliever by a placebo administration. It was however worth noting that the blinded administration of rimonabant, a cannabinoid receptor antagonist, nullified this placebo effect again [122]. These and other studies performed with imaging procedures like Positron Emission Tomography (PET) and functional Magnetic Resonance Tomography (fMRI) could prove that the application of placebo is associated with an activation of neurotransmitter systems which are also responsible for physiological pain processing in the brain [123], [124].

A placebo-induced influencing of central transmitter systems, e.g., endorphines, cannabinoids, cholecystokinin or dopamine and their accompanying brain structures is also observed for other disorders or diseases and considered overall as a neurobiochemical correlate of conditioning and expectation [98], [125]. These neurophysiological measurable changes allow further decoding of individual phenomena which accompany the placebo effect. They however do not represent any general proof of clinical efficacy for placebo.

### Response rate, dosage and effective period

Pharmacological aspects like response rate, dosage and effective period were also studied for placebos. The percentage of patients that show a significant effect as part of a placebo treatment depends on treatment context and kind of disease [93], [126]. Huge variability was also observed for the potency of placebo [98]. A generalisation has not been possible for both parameters, i. e., incidence and potency of placebo effects. A certain reference dose may also apply to placebo: thus it was observed that two placebo tablets can act more strongly than one tablet. Placebo effects are however apparently dependent to a much greater extent on numerous factors from the overall treatment context than verum effects. The complexity of influence factors on the placebo effect also entails that reproducibility of placebo responses is only present if all details of the treatment context are held constant. Even the smallest detail changes, like a name change of the placebo can eliminate the placebo effect [98]. However, not all of these investigations satisfy the above mentioned stringent criteria for determining the placebo effect (see above section “Efficacy contexts”). The effective period of placebo is usually shorter than that of verum, as it was shown for the effect of placebo vs. apomorphine on muscle rigidity in Parkinson’s disease [127].

### Side effects

An important question from the pharmacological area concerns side effects triggered by placebos. Placebos can indeed trigger not only positive but also harmful effects as per expectation [99], [128], [129]. If the negative effects predominate, a placebo becomes a nocebo (“I will be harmful”). Even nocebo effects have an important significance in clinical day-to-day life. They may be involved in adverse drug effects, e.g., occurrence of sexual function disorders in case of betablockers [129].

A principally different risk of adverse effects accompanies the use of real active ingredients, which are consciously administered as “pseudo placebos”. This practice, by no means rare, is naturally afflicted with the inherent risks of side effects of the relevant active ingredients. E.g., if antibiotics are consciously desired to be used for virus infections for their hoped (additional) placebo effects, risks of the development of allergies and resistance exist. 

### Prescription frequency

The use of placebos does not appear to be rare in daily practice. 70 up to about 80% of general practitioners and about 40 to 50% of clinicians or specialist doctors domestically and internationally indicate in different surveys that they have used placebos in the respective previous year [130], [131], [132], [133], [134], which does not allow any conclusion about the percentage of placebo treatments in all or given treatment situations. Primarily phytotherapeutics, homeopathic products and vitamins, but also antibiotics, sedatives and analgesics are prescribed as pseudo placebos here; pure placebos are prescribed much more rarely. According to a study from the USA published in 2008, only 4% of the patients were explicitly informed about the placebo nature of the medication. They were much rather told that the medication may help, but does not harm; it acts in a non-specific or unclear manner [133]. 

## Medicinal CAM and placebo therapies: Pro and Contra arguments

Every patient has a right to the best possible therapy; this motto is mentioned quite often as a core element of a doctor’s ethos and also regarding the fairness in the allocation of medicinal resources. Even if this guiding principle clearly remains an idealisation, considering the limited funds and fallible therapeutists even in affluent societies with publicly financed health care (German social insurance law speaks of “services” that must be “sufficient, purposeful and economical” [135]), it nevertheless sets an important benchmark for treatment standards. If justified or visibly unchangeable external factors, like shortage of funds in the health system, are prejudicial to satisfying this benchmark, this fact can eventually be accepted or influenced politically. If individual doctors are not trained in the latest technique, this fact may be countered by structural measures. However, if their standard of best possible therapy is itself questioned from the perspective of science-oriented medicine, this requires a public and critical debate about ethical, scientific and action-theoretical questions. Precisely this situation appears to be occurring if medicinal products are prescribed without proven (specific) efficacy for the present illness as is the case with CAM procedures and placebo therapies.

What CAM and placebo preparations have in common is that they lack definite evidence of clinically relevant efficacy, although both have access to assessments via the instruments of evidence-based medicine. Their effects may essentially be explained as context effects. For this reason, CAM preparations from the domains of homeopathy and anthroposophy are often classified as impure or pseudo placebos, whereas other preparations, e.g., high potency homeopathic products may more easily be associated with pure placebos [69], [73], [92], [136], [137], [138], [139].

Differences between CAM and placebo can however be found in the theories underlying their prescription. Exponents of CAM procedures generally invoke paradigms established outside science-oriented medicine, while it is attempted to explain the placebo effects on the basis of scientific medicine. It has not yet been sufficiently verified whether CAM preparations act better if both doctor and patient are convinced about a specific healing power of the method, and if they are not administered only as an impure or pseudo placebo.

It has been advocated in a current paper to minimise placebo effects in developing medicines, but to maximise them in the clinical use of effective medicinal products, without however misusing them for using ineffective medicines. It is expected that individual (“customised”) placebo responses will play a bigger role in therapeutic practice in the future while considering genetic disposition, individual medical history and other factors [140]. 

### About the use of CAM preparations

A series of arguments [141] and justifications are proposed in favour of CAM therapies which will be critically tested below.

#### The argument of the holistic approach

Some CAM exponents invoke “holistic form recognition” as a therapeutic principle. In a certain interpretation, its plausibility is immediately evident and it also has its place in science-oriented medicine. “Form recognition” then means a holistic view of symptoms replete with experiences, as experienced nursing staff and doctors practice, who can literally “smell” a diagnosis, before other parameters are compiled. If “form recognition” however simultaneously involves criticism in meaningful “reductionist” diagnostic and therapeutic accesses, as is the case in isolating and exterminating bacteria or viruses as triggers of traditional acute infection illnesses, the principle is dogmatically excessive. The same applies to a supposed “holistic control on bodily regulation systems” or an “upset life force” as it is propagated by other CAM exponents: these mechanisms may sound elucidatory to lay people, but are neither proven at a pathophysiologic level nor in terms of clinical efficacy.

As stressed above, holistic consideration and attention are not specific features of alternative medicine. If there are deficits in science-oriented medicine in this regard, this is unfortunate and must be changed. Such negative developments may however never be used as an argument in favour of an “alternative” therapy approach.

#### The argument of therapy success (“the one who cures is right”)

Curative successes claimed by CAM exponents are generally of a casuistic-anecdotal nature. The conclusiveness of such sporadic and subjective, i.e., “unregulated” observations were quite rightly already questioned by Francis Bacon (1561–1627). His unease led to the requirement for a “regulated experience” (experienta ordinata), i.e., a methodical approach to the planned experiment [142]. Modern Evidence-based Medicine is based on this principle. Precisely the individuality in medicine also emphasized by CAM exponents requires the stochastic approach [143]. The claim of “the one who cures is right” must thus be complemented by methodically clean evidence to prove a causal relation between cause (treatment) and effect (cure) and to prevent the confusion of a “post hoc” conclusion with a “propter hoc” conclusion (see above).

#### The argument of partial dispensability of EbM standards

A series of objections are raised and possible weaknesses are stressed by CAM exponents regarding the orientation of practical medicine to the results of stochastic analyses (EbM) for answering the question of whether a therapy is effective. In addition, it is not rare that CAM exponents deny the competence of science-oriented medicine to evaluate CAM procedures [144].

Dispensing with the maintenance of conventional efficacy evidence in medicine would be a big step backwards which cannot be seriously discussed. But what does it mean to accept a second standard as part of “integration concepts” for CAM methods within established medicine besides the EbM standard, especially that of the (alternative) “internal knowledge”? The first answer is that in actual fact such an addition actually questions the first standard. A detailed answer can be discussed at different levels: 

It is (a) claimed that there are therapeutically relevant effects which cannot be measured with EbM standards. If this is correct, it must however also apply to standards of science-oriented medicine. This would imply that the basic scientific axioms of observability, controllability, repeatability and principal explicability of causal effects and their stochastic implementations would be abandoned.

The doubt is (b) established by the fact that many methods and standards in the daily clinical experience of science-oriented medicine do not satisfy EbM standards so that it is no necessary condition to professionally accept it as established therapeutic measures. This claim is correct. However, it can’t be the basis for arbitrary for efficacy tests, but for an imperative for step-wise checking of all procedures not yet sufficiently evaluated, which would be dismissed in case of negative results. Many CAM exponents however want to prevent precisely this procedure for their therapies.

**Figure 4:**
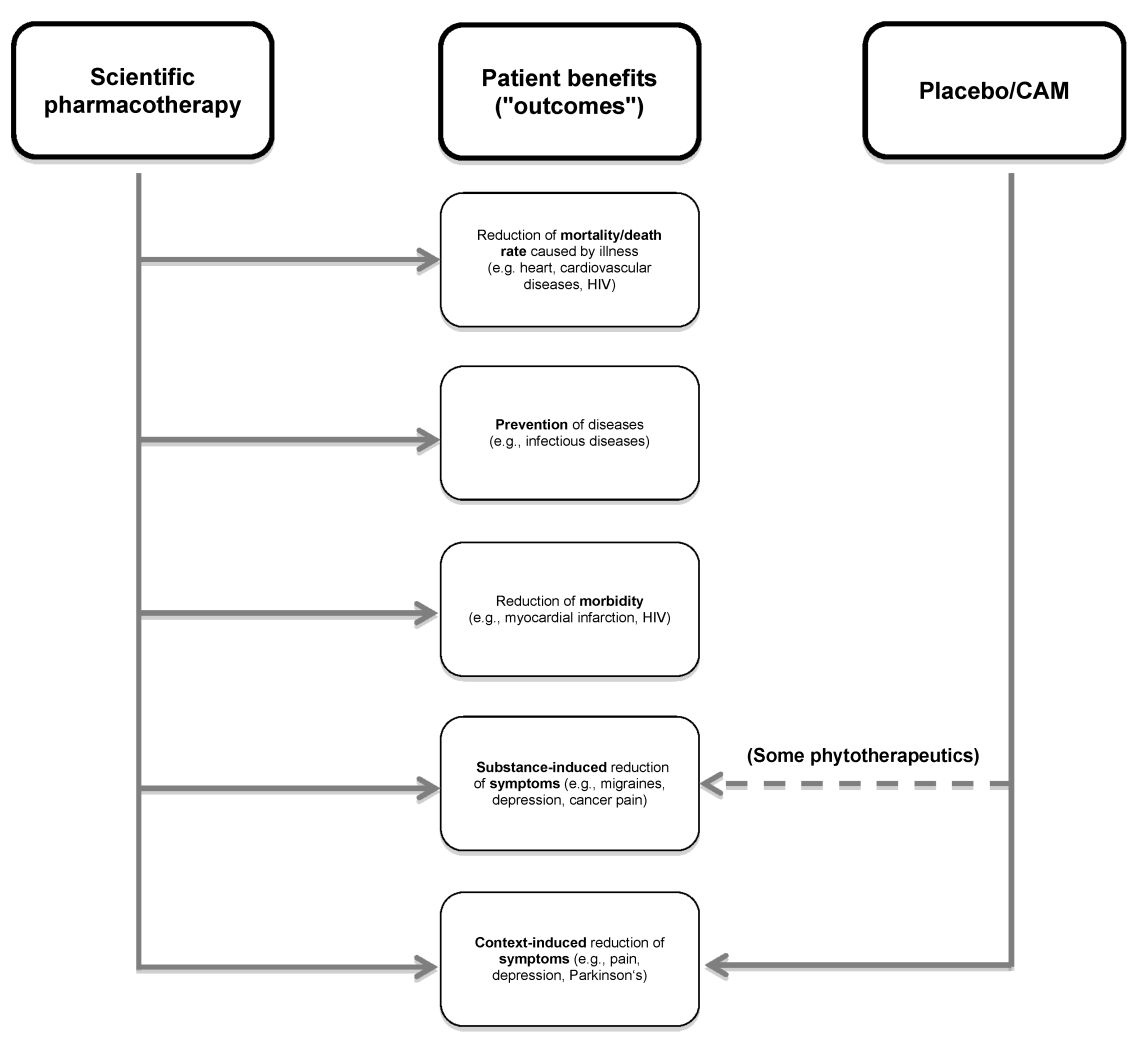
Successes specific to patients of scientific pharmacotherapy in comparison with medicinal procedures of complementary/alternative medicine or placebo administration

Finally, the doubt can be linked to the argument (c) that even science-oriented medicine cannot really explain very many of its curative results (e.g., the effect of lithium as anti-depressive agent; see also Figure 1 (Fig. 1): 1b). This applies especially to complementary medicine that the surrogate parameters cannot (yet?) be determined in therapeutic comparison. This argument now confuses the (desirable, but clinically dispensable) explicability of a curative success with a stochastically conducted efficacy proof (see above) and plays down the fact that parameters of the life extension or health-related quality of life must be utilized for evaluating all therapy procedures, at least the first is clearly measurable for groups. Moreover, we know of no study, which proves the potential for life extension which has been proven for numerous scientific therapy forms, for a CAM measure (Figure 4 (Fig. 4)).

The requirement for being based on evidence applies to medicine overall. Even if it pursues other intervention approaches, CAM may not be excluded from it. Doctors who give up the basic requirement, that apart from individual curative attempts only proven standards with self-evident efficacy chances are introduced or carried out with a view to CAM, open up the keys to irrational medicine. The imperative of science, which requires advancing medical progress by ever stricter checking of previous procedures, is precisely the inverse. If the criterion of testing the efficacy is given up, it becomes difficult to fight against the entry of charlatanry and any possible ideologically founded procedures in medicine.

#### The argument of patient preferences

Demand for CAM procedures by patients and their relatives has increased in the last 20 years (see above). Many CAM measures are considered to be effective and simultaneously, precisely in contrast to science-oriented medicine, harmless, human, holistic, forward-looking and legitimately critical of technology. This especially also applies to “exotic” procedures like those of Traditional Chinese medicine or Ayurveda: their thousands of years old culture, diverse repertoire and claim to individuality suggest that they have also stood the test of time and also offered hopes for a completely different culture group [145].

These evaluations, which are also represented remarkably often by scientifically trained citizens, intellectuals and opinion leaders, are all questionable from the perspective of science-oriented medicine since they consider suitably marketed aspects of the alternative medical practice, however underestimate to what extent these procedures do not satisfy established and practical efficacy standards in this regard. The changed understanding of medicine by the population, which tries to extend the hitherto predominantly indication-related and curative use in the sense of “medicine that fulfils desires” for self-realisation and life planning, has certainly contributed to the increased attractiveness of alternative medical procedures to a large extent [146].

The discussion is moreover rendered difficult by understanding and communications problems, which are not seldom the result of too narrow a commitment against the convenience of undisciplined thinking [147]. The medical historian Tröhler correctly criticises: “The exponents of objectivity, doctors who represent a methodically validated, therapeutic experience, and subjectivity, thus in advance for patients, speak different languages which are mutually becoming ever more difficult to understand [3].” Doctors are however responsible, like everywhere else in medicine, to pursue explanations, instead of easily and conveniently fulfilling the desires of their often insufficiently informed patients.

#### The argument of naturalness and softness

CAM is commonly equated with naturalness and softness of the treatment. This misrepresents the fact that naturalness is in no way a guarantee of harmlessness. Rather, anthroposophics and homeopathic products besides components from plants, animals and salts may contain even thoroughly risky toxicological metals, e. g., arsenic, lead, cadmium and mercury. Reference has already been made above to side effects of complex herbal medicinal products, also caused by the additions of undesired substances. The fact that side effects from this area are rather seldom observed overall would only apply as a relevant pro argument if efficacy was actually proven and a positive benefit-risk ratio justified the use.

#### The argument of patient attention

An undisputed advantage of CAM consists of the mostly much stronger practice of providing attention to the concerned patients. Sufficient time for patient consultations and examinations, engaged listening, communicative competencies and a true and patient interest for the subjective aspects of the illness belong to the self-understanding of every human medicine, but are increasingly explained as the virtually unique feature of CAM. It was shown in a clinical study that clinical improvements in patients with rheumatoid arthritis are associated not with the homeopathic medication, but with elaborate homeopathic consultations [148].

Science-oriented medicine, on the contrary, has precisely neglected this doctor’s virtue in the decades of its technical equipment, and must undoubtedly set new courses, extend its knowledge and press for other time and economic boundary conditions, which enable a further revaluation of the “talking medicine” (see above).

It however needs to be stressed once again that linking patient attention to CAM and delinking it from science-oriented medicine is not associated with obeying any necessary rule or necessary different human being images, roles of doctors or notions of illness. Individual patients and their cares must be the centre of all activities also for doctors who consider themselves to be committed to science-oriented medicine. CAM procedures cannot in any way be justified only for these reasons, which nonetheless are a plausible partial explanation for the huge demand for CAM by patients.

#### The factuality argument

Patients may be encouraged in their demand for CAM by the fact that trained doctors offer these measures to an increasingly greater extent and thus effectively “ennoble” them. These doctors however possibly predominantly react to the observed patient preferences with their offers. Thus, not only a problematic spiral, but additionally a sneaking habituation to the non-observance of scientific medical standards, is created [149]. The existing integration of CAM procedures in the training and continuing education of doctors in Germany is also problematic from this point of view, e.g., by the additional designation of “homeopathy”, due to which they are given considerable pseudo legitimacy [1], as well as due to the reimbursement of costs by health insurers.

#### The argument of low costs

It is not seldom stated in favour of CAM that with regard to existing or supposed indications these procedures are for the most part considerably less expensive than standards of science-oriented medicine. Although cost savings in the health system are undoubtedly important and will continue to be so in the future, any isolated consideration and evaluation of costs still appears meaningless. Costs incurred for conducting ineffective procedures are always too high. A comprehensive systematic review for the cost effectiveness of CAM procedures, which also considered the design of the underlying effectiveness studies, found mostly additional costs and cost reductions in about 30% of the studies; in this regard, reference was made to the substantial heterogeneity of the quality of studies, as in other reviews [150], [151]. Integrating CAM procedures without consideration of their efficacy because they are more cost-effective than science-oriented medicine and because this introduction therefore does not carry much weight or could lead to savings does not correspond to the social law and ethical requirements of medical care. Moreover, procedures of CAM are commonly applied in addition to existing therapy.

### About the use of placebo preparations

The prescription of placebo preparations in therapeutic practice and the few recommendations existing for this purpose ignore formal criteria and essential principles of scientifically justified medical therapy.

#### The “formal” criterion for approval

Recommendations for pharmaceutical prescriptions are usually associated with several conditions. These entail formal requirements derived from the properties of medicinal products and created using study results for the approval. Approval applies to specific, tested fields of application (indications). No approved preparations with a “placebo indication” exist. The more commonly practiced use of active ingredients in the sense of impure or pseudo placebos, e.g., antibiotics in case of non-bacterial infections, thwarts the approved indication. It is moreover fraught with dangers (see above).

#### Efficacy and benefit proof

Intentional use of preparations with inert contents or in idle, unapproved indication requires special justification. The best possible evidence of efficacy and benefit of a therapy consist of clinically relevant results of randomised controlled studies and corresponding meta-analyses. Contrary to many modern medicinal products, no such studies are available for placebo preparations. No clinically relevant advantage of a placebo medication was found for objectively assessable end-points in a multiply updated meta-analysis of three-armed studies, which also enabled a comparison of placebo for “non-treatment” besides the verum [94], [107], [109], [152]. 

Furthermore, sufficient data about different parameters like response rate, potency, effective period, and reproducibility, are missing. Robust evidence which would authorise the general recommendation of placebo preparations therefore does not exist [136], [152], [153]. 

#### Undesired effects

Nocebo effects may occur depending on context. Since pseudo placebos are used more often than pure placebos as per epidemiological data, side effects depending on substance need moreover to be taken into account.

#### Ethical concerns 

Explanation and information with the objective to design the treatment by mutual agreement with the patients are part of the doctor’s obligations of every pharmacotherapy. Since placebo effects benefit from the subjective confidence in the administered medication, patients are generally not told about the “exclusively” placebo nature, precisely not to take away from the suggestive and expectation effects (by or of a supposedly authentic treatment procedure). Many authors, but also committees of professional medical associations see a serious and potent ethical problem in this delusion about the nature of medication besides the objection of missing efficacy evidence [94], [98], [136], [153], [154], [155], [156], [157]. More placebo friendly positions, on the other hand, as currently represented by the German Medical Association [92], are justified with the argument that placebo administrations do however represent the most appropriate patient treatment in certain constellations.

Hopes of circumventing this dispute are based on some more recent studies about the efficacy of “open” placebo treatments. Thus, results of some smaller studies on psychiatric diseases such as anxiety disorders and ADHS, as well as irritable bowel syndrome, a disease observed with subjective measurement parameters and episodically occurring migraines, points to the fact that a placebo effect may also possibly occur if the patient was previously told about the placebo character of the medication [112], [158], [159], [160]. A recommendation to “openly” administer placebos in therapeutic practice cannot however (yet) be derived from these studies, which are partially fraught with methodical shortcomings [159], [160] or present results of a pilot study in a singular indication which are to be confirmed [158]. 

The above considerations are very generally related to the administration of hidden therapeutic placebos. However, from our viewpoint, there is an important specific problem if doctors use CAM measures of all things as hidden placebos. Such a practice is currently occurring to a large extent whenever doctors recommend CAM measures as potentially effective treatments, although they do not have any specific effect as per scientific standards (see above). The explanation for this practice is evident: either therapeutists themselves, uninformed or sceptical to science, believe in the efficacy of their measures or they consciously use them as placebos. In the second case, the CAM affinity of many patients and the widespread “charisma” of these standards promise especially reliable placebo effects. At the same time, however, and here we see a serious problem: there is an (apparent) recognition and even ennoblement of ineffective CAM methods by exponents of science-oriented medicine, who finally question its standards and the exponents of such standards (also see [161]). We consider such signals to be highly problematic.

#### Importance of context effects (“contextual healing”)

The discussion on the importance of non-specific effects of the overall therapeutic environment is commonly narrowed to the administration of an administrable placebo preparation. It has long been known, in this regard, that “therapeutic manipulations, impressive equipment, the entire environment, but primarily the doctor’s personality and his/her association with the patients: all these elements in the individual highly complex patient/doctor relationship, are designated by Martini as the magical part of therapy,” also contribute to treatment success of conventional medicines [16]. It is attempted to clarify this “magical” part better with neurobiological, epidemiological and clinical studies.

According to the results of the already cited Cochrane Reviews of Hrobjartsson et al., also the rather limited unassured advantages of a placebo medication for most indications in comparison with untreated groups which experienced no medication, but comparable attention as part of the study, promote the fundamental importance of the overall treatment context [94], [153]. Thus, the formation of the doctor-patient relationship, although examined insufficiently until now using clinically relevant parameters, certainly holds potential to improve results of therapies through conscious use of non-specific effects [112], [140].

A possible catalogue of behaviours and discussion techniques for inducing non-specific positive effects in therapeutic practice is shown in Table 4 (Tab. 4).

These properties basically belong to the indispensable skills of each and every doctor, especially the general practitioner. In times of high-technology and measurement result-oriented medicine, under time and performance pressure, such qualities are unfortunately often neglected. Particularly in functional and somatoform diseases the general practitioner’s consultation or psychotherapeutic treatment [162] needs to be preferred over medication, even with placebos, depending on the severity of the illness.

The relationship of the use of specific and non-specific measures related to efficacy, side effects and costs should moveover be reconsidered in case of chronic diseases, especially in functional disorders and advanced incurable physical diseases. For example, increasingly greater time resources of the doctor and financial resources of the health system are consumed for limited gains in life time through specific oncological treatments with potentially higher risks for the patients [163]. The time consumed for specific measures is not available for patient discussions, e.g., about the psychosocial consequences of the illness. The current fee structure of medical services incorrectly gives incentives for implementing specific measures and omitting counselling in somatic medicine [164]. The talk with patients including discussing life impairment and impending death is transferred to the psychosocial specialities or even to CAM with the risk of loss of orientation.

In order to prevent a further “exodus” of patients with functional disorders and chronic physical ailments to the area of complementary and alternative medicine, stronger consideration and activation of non-specific action factors like time, understanding and compassion are necessary. Greater weight must be placed on learning interpersonal skills (empathy, conversation) in medical studies and in the doctor’s training and continuing education. The most recent inclusion of doctor’s conversation as a study and test subject in the regulation for licensing doctors is an important step in this regard [165].

The task of professional organisations and the legislator is to create the administrative, organisational and financial pre-conditions for patient care that allow more intensive patient attention without loss of quality of care. Efforts to promote the requirement for increased attention and stronger consideration of patient desires through facilitating the access to therapeutic standards, whose only effect is based on context effects, but not on evidence of efficacy of scientific medicine, do not however offer any acceptable solution. In this regard, one is guilty of arbitrariness in choosing the paradigm and deviating from scientifically justified medicine, under whatever guise it may be. “Striving for harmony may be politically, even professionally, opportune, but cannot be sustained from a point of view of scientific theory” [16].

In this regard, doctors have the responsibility to strive for explanation, training and fortifying their patients for a responsible contact with their own health and illness, instead of comfortably and pseudo-empathically “letting the customer be king” and even satisfying wishes of uninformed patients (“requested medicine”) frivolously. In general, the danger is additionally present that a frivolous readiness to administer placebos strengthens the widespread behaviour that every illness or sensitivity disorder must absolutely be treated with medicinal measures (there is a pill for every ill; disease mongering) [136], [152], regardless of practical benefit/harm considerations, almost as a substitute for care or required explanation about the nature of the disorder.

## Summary and recommendations

1. Ideal standard for patient care is the best possible treatment. In medical therapy, this is the sum of the specific effects of a demonstrably effective drug and the “non-specific” positive effects of the overall therapeutic context for the correspondent existing indication.
2. It is of paramount importance to recommend therapeutic procedures based on a plausible efficacy model whose specific effects and positive benefit-risk ratio could be proved with the methods of Evidence-based Medicine. Use of a treatment procedure is possible if either a plausible, i.e., scientifically explicable efficacy model or relevant results from clinical studies is/are available. Therapeutic procedures for which both conditions are missing are to be advised against.
3. Even CAM or placebo procedures are essentially amenable to efficacy testing with instruments of Evidence-based Medicine.
4. Medicinal products of complementary and alternative medicine (CAM) are founded with paradigms of pre- or non-scientific medicine in many cases.
5. Evidence of a clinically relevant efficacy as per the standards of evidence-based, science-oriented medicine, which could justify therapeutic use, is not generally available for CAM methods, and also not for placebo medication. Their effects are predominantly conditioned by treatment context. The patient needs to be informed about this.
6. A multitude of negative study results for the efficacy of medicinal CAM procedures question the respective methods on the whole, so that in this regard other research projects are commonly not justifiable.
7. As in science-oriented medicine, consequences should also be drawn for CAM treatments from available results of valid studies. Medicines and methods, whose efficacy does not exceed that of placebos, should not have any place in therapeutic practice.
8. Combined use of scientifically justified and complementary medicine procedures as part of the so-called integrative medicine negates contradictory paradigms and contradicts the principle according to which every component of a therapy has to provide an individual and provable contribution.
9. Utilizing the charisma of CAM measures to consciously employ them as just therapeutic placebos ennobles the scientific scepticism of many CAM exponents fatally.
10. Not every treatment requires medication. A placebo prescription promotes medicalisation with questionable efficacy. Primarily in case of functional and somatoform disorders, depending on the degree of severity, non-medicinal measures and a general practitioner’s, or, if required, also a guideline compliant psychotherapeutic/psychosomatic treatment can be recommended.
11. Doctor’s empathy and attention are essential elements of patient-oriented medicine as a part of scientifically justified treatment. They are also partly scientifically attestable and form the basis for the optimal efficacy of science-oriented medicine. This task which cannot easily be provided in the doctor’s daily routine should not be delegated to CAM or a placebo administration due to confidence in context effects.

## Notes

### Competing interests

The authors declare that they have no competing interests.

### Authorship

The names of authors are listed in alphabetical order. All aforesaid authors are current or former (Leidl R) members of the Drug Commission of the German Medical Association.

### Acknowledgements

Our acknowledgements for criticism and suggested approvements go to

- Dr. phil. Daniel R. Friedrich, Münster
- PD Dr. med. Jutta Hübner, Berlin
- Prof. Dr. med. Dietrich Höffler, Weiterstadt
- Prof. Dr. med. Dr. phil. Peter Hucklenbroich, Münster
- Prof. Dr. med. Rudolf Wilhelm Christian Janzen, Frankfurt/M.
- Prof. Dr.med. Thomas Kühlein, Erlangen
- PD Dr. med. Martina Pitzer, Karlsruhe
- Prof. Dr. med. Dr. h.c. Wolfgang Rascher, Erlangen
- Prof. Dr. med. Dr. phil. Heiner Raspe, Lübeck
- Dr. phil. Jan-Ole Reichardt, Münster
- Prof. Dr. med. Ulrich Schwabe, Heidelberg
- Prof. Dr. med. Gabriela Stoppe, Basel
- Prof. Dr. rer. nat. Hans-Joachim Trampisch, Bochum
- Prof. Dr. med. Jürgen Windeler, Köln

- Translation of the German script: Sandra Huth, Cologne, Germany

## Figures and Tables

**Table 1:**
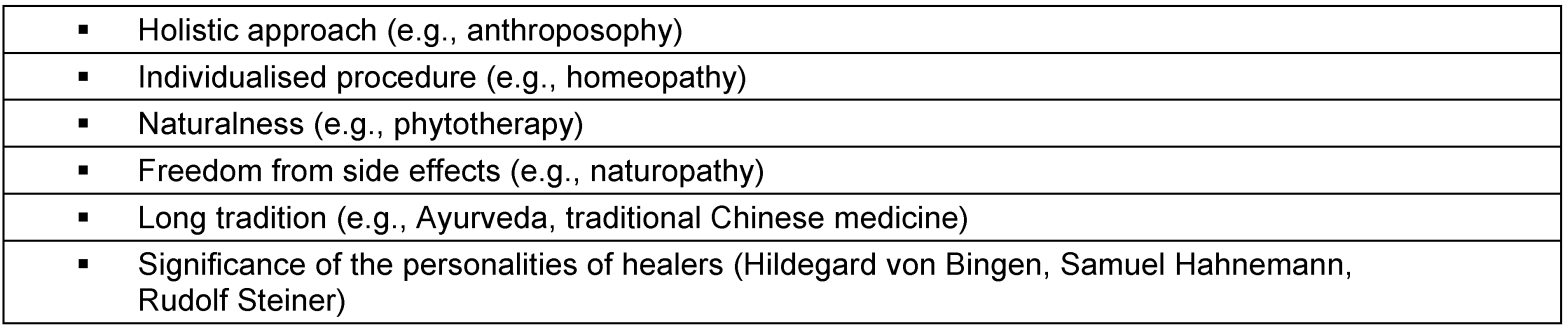
Different criteria for characterising complementary or alternative medical methods

**Table 2:**
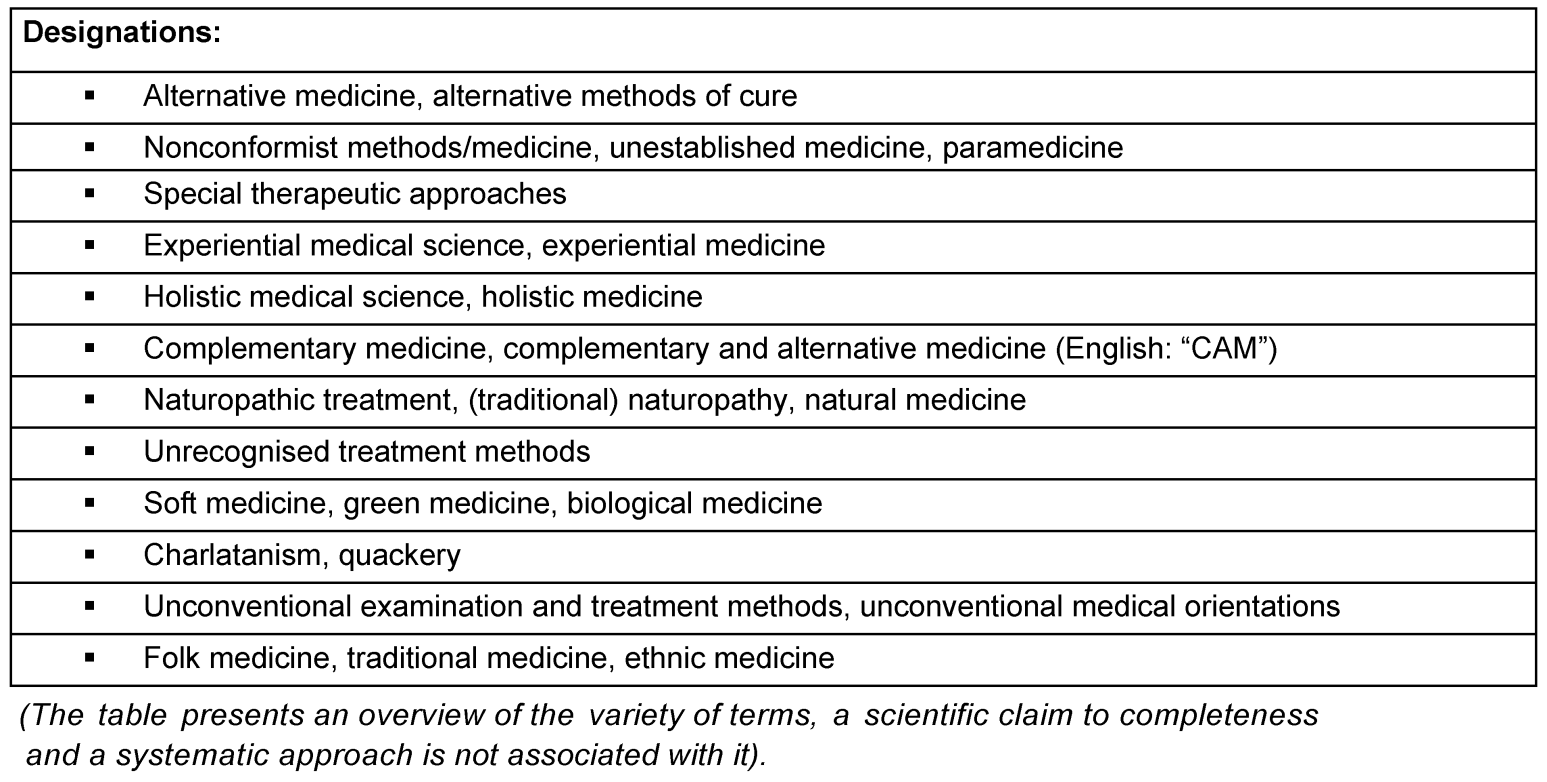
Designations for alternative therapy and diagnosis procedures, according to [81]

**Table 3:**
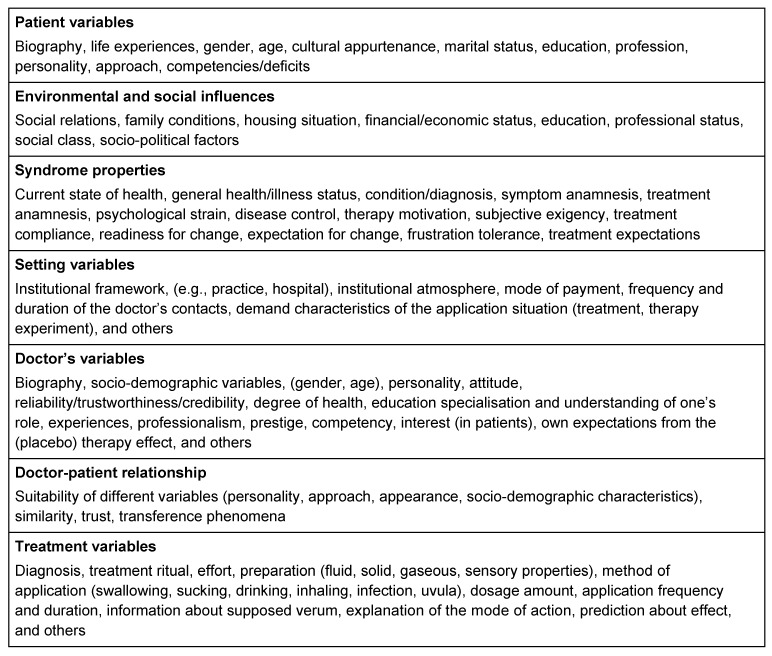
Possible context factors according to Windeler [166]

**Table 4:**
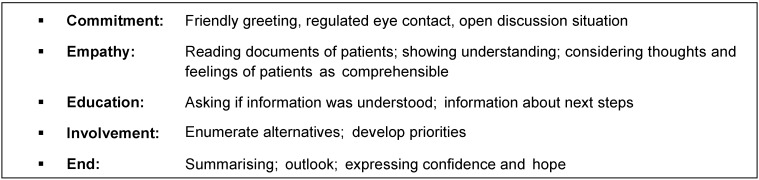
Five behaviours and discussion techniques for inducing non-specific positive effects according to Jamison [167]
